# UAV-Based Estimation of Tea Leaf Area Index in Mountainous Terrain: Integrating Topographic Correction and Interpretable Machine Learning

**DOI:** 10.3390/s26072218

**Published:** 2026-04-03

**Authors:** Na Lin, Jian Zhao, Huxiang Shao, Miaomiao Wang, Hong Chen

**Affiliations:** Institute of Digital Agriculture, Fujian Academy of Agricultural Sciences, Fuzhou 350003, China; linna30@163.com (N.L.); zhaojian@faas.cn (J.Z.); shx9601@163.com (H.S.); wangmm.16b@igsnrr.ac.cn (M.W.)

**Keywords:** leaf area index, UAV, topographic correction, machine learning, SHAP, tea plantation, canopy texture

## Abstract

Leaf Area Index (LAI) is a fundamental parameter for characterizing the growth of tea (*Camellia sinensis* L.). However, in rugged mountainous regions, the combined effects of topographic relief and canopy structural heterogeneity severely constrain the accuracy of UAV-based multispectral LAI retrieval. This study develops an integrated framework combining topographic correction with interpretable machine learning to improve LAI estimation. We utilized a UAV multispectral dataset collected during the peak growing season from a typical tea-growing region in Fujian Province, China (altitude range: 58–186 m), comprising a total of 90 samples. Three topographic correction methods, including Sun–Canopy–Sensor (SCS), SCS with C correction (SCS+C), and Minnaert+SCS, were evaluated in combination with Linear Regression (LR), Decision Tree (DT), Random Forest (RF), and Extreme Gradient Boosting (XGBoost) models. Results indicated that the SCS+C algorithm outperformed other methods by effectively accounting for direct and diffuse radiation components, thereby reducing topographic dependence while maintaining radiometric consistency across heterogeneous surfaces. The XGBoost model combined with SCS+C correction achieved the highest performance (R^2^ = 0.8930, RMSE = 0.6676, nRMSE = 7.93%, MAE = 0.4936, Bias = −0.0836). SHapley Additive exPlanations (SHAP) analysis revealed a structure-dominated retrieval mechanism, in which red-band textural features (Correlation_R) exhibited higher importance than conventional vegetation indices. Compared with previous studies that primarily focus on either topographic correction or model development, this study provides quantitative insights into the underlying retrieval mechanisms. This framework improves the precision of tea LAI retrieval in complex terrains and provides a robust methodological basis for digital management in mountainous agriculture.

## 1. Introduction

Tea (*Camellia sinensis* L.) is one of the most significant perennial cash crops globally, with a total production exceeding 32.7 million tonnes in 2024 [1]. Its growth dynamics and physiological status are critical determinants of both yield formation and the secondary metabolites that define leaf quality. Leaf Area Index (LAI), defined as the total one-sided green leaf area per unit ground surface area, is a fundamental biophysical parameter describing vegetation canopy structure [2,3]. Accurate LAI estimation is essential not only for assessing photosynthetic capacity but also for supporting carbon cycle analysis, nutrient management, and precision cultivation in tea production systems [4,5,6].

Traditional ground-based LAI measurements, including destructive sampling and optical instruments, are constrained by low efficiency and limited spatial representativeness. Although satellite remote sensing enables large-scale monitoring, its application in fragmented agricultural landscapes is often hampered by coarse spatial resolution, inflexible revisit cycles, and atmospheric interference. In heterogeneous environments, pixel mixing effects further degrade retrieval accuracy [7,8]. In contrast, Unmanned Aerial Vehicle (UAV) remote sensing has emerged as an effective tool for plot-scale crop phenotyping, offering ultra-high spatial resolution, flexible acquisition schedules, and cost-effective data collection [9,10].

China, the world’s largest tea producer, primarily cultivates tea in hilly and mountainous regions, where complex terrain poses a major challenge to quantitative remote sensing. Previous studies have reported that topographic effects can introduce substantial uncertainties in LAI retrieval, with relative errors or RMSE increases exceeding 50% under complex terrain conditions [11,12]. Variations in slope and aspect cause spatially heterogeneous illumination conditions, resulting in radiometric inconsistency and weakening the physical linkage between spectral reflectance and canopy biophysical properties [13]. To mitigate these effects, various topographic correction models have been developed. Common approaches include empirical methods such as C-correction, Minnaert correction, and other semi-empirical models, which aim to normalize illumination differences [14,15,16]. The Sun–Canopy–Sensor (SCS) model [17] corrects reflectance based on geometric relationships between solar incidence and terrain orientation but often suffers from overcorrection in shaded areas. The SCS+C model [18] introduces an empirical parameter to account for diffuse radiation, thereby reducing illumination-induced bias in complex terrain. The Minnaert+SCS approach [19,20] further combines non-Lambertian surface assumptions with geometric correction to enhance model robustness. Nevertheless, the effectiveness of these algorithms for UAV-based monitoring in rugged tea-growing regions has not been fully validated. In such plantations, the combination of steep slopes and artificial ridge-furrow structures introduces fine-scale topographic complexity, leading to pronounced illumination variations. This heterogeneity is particularly problematic for high-resolution UAV sensors, where shadowing and occlusion become more apparent. Consequently, a comprehensive assessment is required to determine the optimal approach for ensuring radiometric consistency across these heterogeneous terrains.

Regarding modeling methodologies, Machine Learning (ML) algorithms have emerged as powerful tools for LAI inversion due to their demonstrated capabilities in high-dimensional nonlinear modeling. ML-based UAV remote sensing has been extensively validated across various vegetation types, ranging from staple cereal crops like rice and wheat [21,22] to structurally complex forest canopies [23]. For instance, Yuan et al. [24] evaluated the performance of Random Forest (RF), Artificial Neural Network (ANN), and Support Vector Machine (SVM) models for soybean LAI inversion using UAV hyperspectral imagery; Liu et al. [25] assessed maize LAI inversion accuracy employing Light Gradient Boosting Machine (LGBM), Gradient Boosting Decision Tree (GBDT), Random Forest (RF), and Extreme Gradient Boosting (XGBoost) models based on UAV multispectral data. These studies collectively demonstrate the versatility of ML in capturing biophysical parameters across diverse crop geometries. Nevertheless, model performance is highly dependent on the stability of input features, and terrain-induced radiometric distortions can further amplify prediction uncertainty [26], especially in rugged environments. Moreover, the “black-box” nature of many ML models limits physical interpretability, reducing confidence in their applicability across varying environmental conditions. The interpretable machine learning approaches, such as SHapley Additive exPlanations (SHAP) [27], have recently been introduced in agricultural remote sensing to quantify feature contributions and improve model transparency. Previous studies have applied SHAP to analyze the relative importance of spectral indices, texture features, and environmental variables in crop parameter estimation and yield prediction, demonstrating its effectiveness in revealing underlying model mechanisms [28,29,30]. However, the application of SHAP in LAI inversion under complex terrain conditions remains under-researched, particularly for high-resolution UAV imagery in mountainous agricultural systems.

To address these challenges, this study focuses on typical mountainous tea plantations in Fujian Province, China. We hypothesize that integrating rigorous topographic correction with interpretable ensemble machine learning will significantly improve LAI retrieval accuracy compared to using uncorrected data. To test this hypothesis, we develop an integrated framework combining topographic correction, machine learning, and model interpretability. Specifically, we (1) systematically evaluate the performance of SCS, SCS+C, and Minnaert+SCS correction methods using correlation and dispersion metrics; (2) compare the LAI retrieval accuracy of Decision Tree, Random Forest, and XGBoost models based on multi-source features, including spectral bands, vegetation indices, and texture metrics; and (3) employ SHapley Additive exPlanations (SHAP) to quantify feature contributions and elucidate the dominant mechanisms underlying LAI prediction. This study aims to provide a robust methodological reference for precision monitoring and digital management of tea plantations in complex mountainous environments.

## 2. Materials and Methods

### 2.1. Study Area and Data Acquisition

#### 2.1.1. Study Area

The study area was established in a tea plantation within the Tea Research Institute of the Fujian Academy of Agricultural Sciences, located in Shekou Town, Fu’an City, Fujian Province, China. The region is characterized by a subtropical humid monsoon climate, with a mean annual temperature ranging from 17 to 25 °C and annual precipitation between 1700 and 2050 mm, concentrated primarily from March to June. The soils are predominantly deep red and yellow soils with a thickness exceeding 1 m, moderate fertility, and pH values ranging from 5.0 to 6.5. These hydrothermal and edaphic conditions provide a favorable environment for tea cultivation.

The site functions as a tea germplasm nursery comprising more than ten cultivars, primarily categorized into large-leaf and medium-leaf types (e.g., Fuyun series, Baiya Qilan, Maoxie, Jinmudan, and Huangguanyin). Pronounced inter-varietal differences in canopy structure and leaf area index (LAI) generate a broad structural gradient, offering representative conditions for LAI model calibration and independent validation across heterogeneous planting materials. The study area is characterized by typical low-mountain and hilly terrain, with elevations ranging from 58 to 186 m above sea level. Variations in slope and aspect generate spatial differences in illumination conditions and canopy reflectance. In addition, the ridge-based row cultivation system of tea plantations creates systematic micro-topographic undulations between ridge tops and inter-row spaces, increasing fine-scale variability in surface geometry under high-resolution UAV observations.

**Figure 1:**
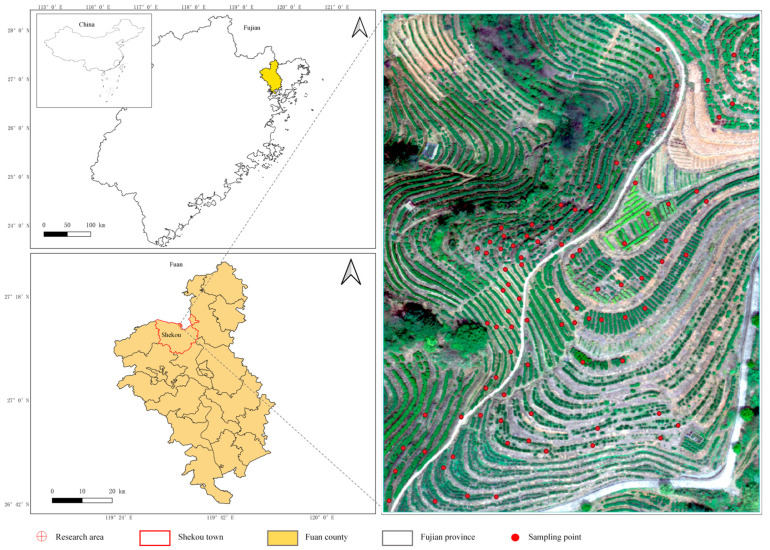
Location of the study area and distribution of sampling points.

**Figure 2:**
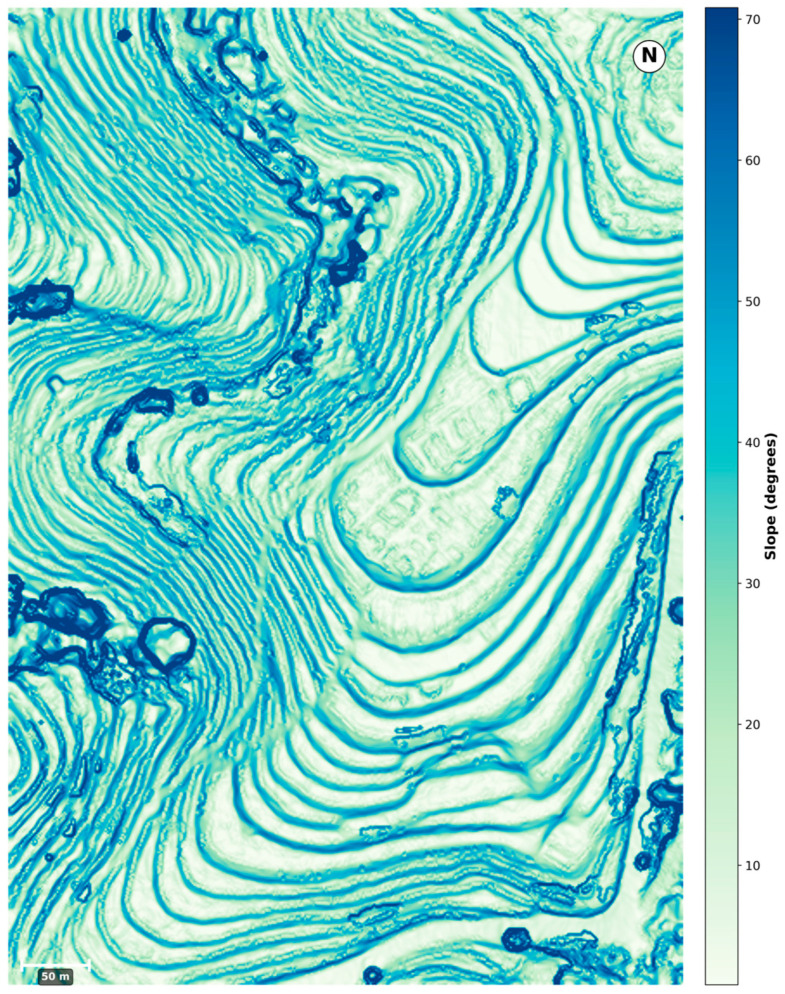
Slope map.

Overall, the combined effects of cultivar heterogeneity and multi-scale terrain variation provide a representative and physically meaningful setting for evaluating UAV-based LAI retrieval and assessing the performance of topographic correction models under realistic tea plantation conditions. The location of the study area and the distribution of sampling points are shown in Figure 1, while the high-frequency spatial transitions of these micro-topographic gradients are explicitly visualized in the slope map (Figure 2).

#### 2.1.2. UAV Image Acquisition and Pre-Processing

UAV-based multispectral imagery was acquired using a DJI Phantom 4 Multispectral (P4M) platform (DJI Technology Co., Ltd., Shenzhen, China). The P4M is an integrated UAV–sensor system with a total takeoff weight of approximately 1.49 kg, requiring no additional payload mounting. It is equipped with one RGB camera and five multispectral sensors: Blue (450 ± 16 nm), Green (560 ± 16 nm), Red (650 ± 16 nm), Red-edge (730 ± 16 nm), and Near-infrared (840 ± 26 nm). The multispectral sensors have a field of view (FOV) of approximately 84°. Data acquisition was conducted on 3 April 2024, corresponding to the vigorous growth stage of tea plants, under stable, cloudless, and windless conditions. Such conditions are preferred to minimize illumination variability and canopy motion, thereby reducing radiometric noise. Flights were performed at an altitude of 200 m, with 80% forward and side overlap, resulting in a ground spatial resolution of 0.06 m. The flight missions were planned using DJI GS Pro software (version 2.0.17) with a predefined grid pattern, and the flight speed was maintained at approximately 2 m/s to ensure sufficient image overlap and to avoid motion-induced image smearing, which was considered negligible under these conditions.

Standard reflectance calibration panels (DJI Phantom 4 Multispectral Calibration Panel, ~50% reflectance) were captured before each flight and used as inputs for radiometric calibration to generate reflectance imagery. Image preprocessing, including radiometric calibration, image alignment, dense point cloud generation, and orthomosaic construction, was performed using DJI Terra software (version 4.0.10). Given the low flight altitude of UAV platforms, atmospheric effects were considered negligible, and no additional atmospheric correction was applied.

The Digital Orthophoto Map (DOM) and Digital Surface Model (DSM) were generated through photogrammetric processing based on image matching and 3D reconstruction within the DJI Terra software environment. The DOM and DSM were georeferenced using the onboard RTK-GNSS positioning system, enabling centimeter-level spatial accuracy without the use of ground control points (GCPs). Lens distortion was automatically corrected during the image processing workflow. A ground-filtering point cloud classification algorithm [31] was applied to remove surface information from vegetation and man-made structures, thereby deriving a high-precision Digital Elevation Model (DEM) for subsequent topographic correction modeling.

#### 2.1.3. Ground Data Acquisition and Processing

To obtain reference values of tea LAI, a stratified sampling strategy was adopted to ensure representative coverage of variations in elevation, slope aspect, and cultivar types across the study area, while considering practical constraints in field data collection. A total of 90 tea plants were selected as representative samples. The sampling was designed to capture variability in canopy structure and LAI across different terrain conditions and tea cultivars, ensuring coverage of the main sources of spatial and biological variability within the study area.

LAI was determined using the Specific Leaf Weight (SLW) method. Although the SLW method may be influenced by cultivar-specific leaf traits and requires destructive sampling, it remains a widely accepted and reliable approach for field-based LAI estimation [32,33]. The SLW-based LAI was calculated as:

*LAI* = (*W*_2_/*W*_1_) × (*S* × *M*)/*A* (1)

where *W*_1_ is the reference leaves mass (g), *W*_2_ is the total mass of all leaves collected from a representative branch(g), *S* is the total area of the reference leaves (m^2^), *M* is the total number of stems per plant, and *A* is the horizontal projection area of the tea canopy (m^2^).

Field data collection and processing were conducted as follows.

1. Spatial Positioning: The spatial coordinates of each sampled plant were recorded using an RTK-GPS system with an accuracy of ±0.01 m. Each plant was measured three times, and the average value was used as the final coordinate.
2. Canopy Measurement: The major (*d*_1_) and minor (*d*_2_) canopy diameters were measured using a tape measure (±1 mm accuracy). Each diameter was measured twice and averaged. The horizontal canopy projection area (*A*) was calculated assuming an elliptical shape:

*A* = π × (*d*_1_/2) × (*d*_2_/2) (2)

3. Stem Counting: The total number of stems (*M*) per plant was manually counted by two investigators. If the difference between counts exceeded 5%, the measurement was repeated.
4. Sampling: Three representative stems per plant were selected based on canopy position and stem characteristics. All leaves from these stems were collected, labeled, and transported for laboratory analysis.
5. Laboratory Analysis: Ten leaves were randomly selected from each sample as reference leaves, and their area (*S*) was measured using the Petiole Pro mobile application (Petiole LTD, https://www.petiolepro.com/). The measurements were calibrated using a reference scale provided by the application to ensure accurate spatial scaling and minimize perspective distortion. The reference leaves (*W*_1_) and total leaves from the sampled stems (*W*_2_) were oven-dried at 105 °C for 30 min to deactivate enzymes, followed by drying at 80 °C to constant weight (±0.001 g) to ensure stable dry mass measurement.

The LAI values ranged from 1.04 to 9.45, with a mean of 3.81 ± 2.12 and a median of 3.16, indicating moderate dispersion and a slightly right-skewed distribution. The coefficient of variation (55.72%) suggests substantial variability in canopy conditions across the study area.

### 2.2. Methods

**Figure 3:**
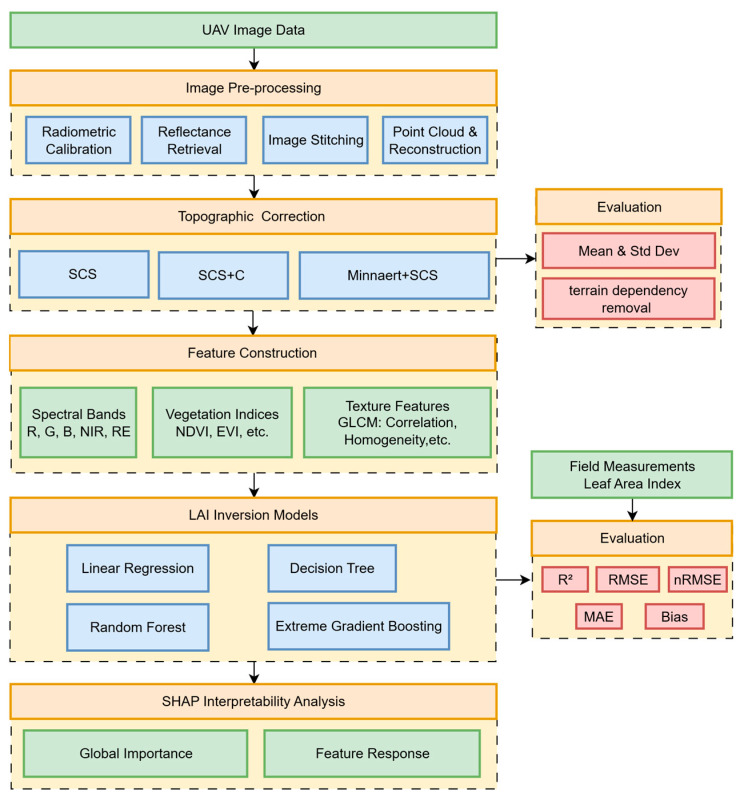
Research framework.

The research framework is shown in Figure 3, consisting of five main stages: data acquisition and preprocessing, terrain correction, feature extraction, model construction, and interpretability analysis.

#### 2.2.1. Topographic Correction

In mountainous remote sensing applications, topographic correction is a critical preprocessing step for mitigating terrain-induced illumination effects and improving the accuracy of quantitative reflectance analysis. In this study, three representative algorithms were selected and systematically evaluated for their applicability to low-stature tea plantation canopies under complex hilly terrain conditions. These models were selected to represent progressively increasing model complexity and illumination adaptability.

(1) SCS Algorithm

**Figure 4:**
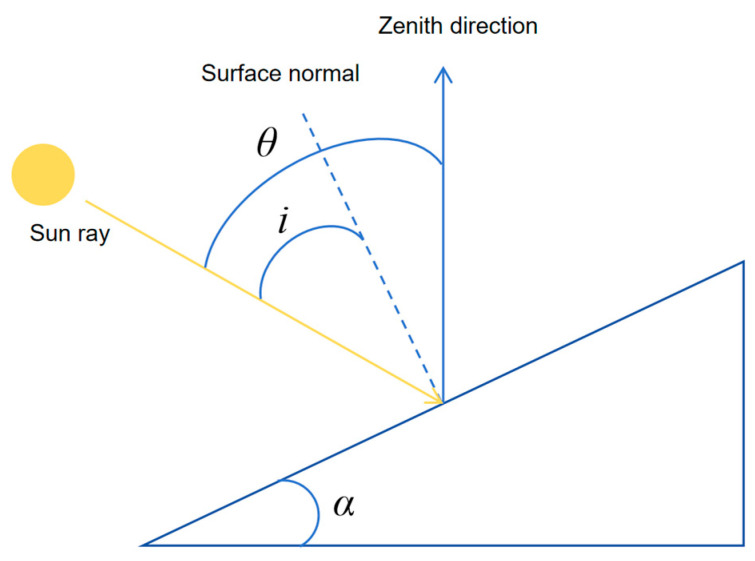
Schematic diagram of the geometric relationships among solar zenith angle (θ), terrain slope angle (α), and solar incidence angle (i) in the topographic correction model.

The Sun–Canopy–Sensor (SCS) algorithm is based on the sun–canopy–sensor imaging geometry. It incorporates topographic slope and aspect to calculate the effective incident angle for radiometric correction [17]. The SCS correction is expressed as:

*ρ_c_* = *ρ* × (cos *α* × cos *θ*)/cos *i* (3)

where *ρ_c_* and *ρ* represent the surface reflectance after and before topographic correction, respectively, with *ρ* derived from radiometrically calibrated UAV imagery. *α* is the terrain slope angle (°), *θ* is the solar zenith angle (°). The geometric relationships among the key angular parameters (*α*, *θ*, and *i*) are illustrated in Figure 4. The cosine of the effective solar incidence angle (cos *i*) is calculated as:

cos *i* = cos *θ* × cos *α* + sin *θ* × sin *α* × cos(*φ* − *β*) (4)

where *φ* is the solar azimuth angle (°), and *β* denotes the terrain aspect (°). Terrain slope and aspect were derived from the UAV-based DEM. In this study, the solar zenith angle was 23.3° and the solar azimuth angle was 157.8° (noon on 3 April 2024).

(2) SCS+C Algorithm

The SCS+C algorithm extends the SCS framework by introducing an empirical parameter C to compensate for residual radiometric differences between sunlit and shaded slopes [18]. This modification relaxes the assumption of purely Lambertian surface reflectance and enhances correction robustness under heterogeneous illumination conditions. The correction formula is given by:

*ρ_c_* = *ρ* × (cos *θ* × cos *α* + *C*)/(cos *i* + *C*) (5)

The parameter *C* is estimated for each spectral band using linear regression between reflectance (*ρ*) and the cosine of solar incident angle (cos *i*), reflecting the regulation capability for topographic illumination differences.

*ρ* = *A* × cos *i* + *B*, *C* = *B*/*A* (6)

(3) Minnaert+SCS Algorithm

The Minnaert+SCS algorithm incorporates the photometric constant k from the Minnaert correction method into the SCS framework, thereby accounting for anisotropic surface scattering effects [19,20]. The correction is formulated as:

*ρ_c_* = *ρ* × (cos *θ*/cos *i*)^*k*^ × cos *α* (7)

The empirical parameter *k* is estimated via log-linear regression and reflects the degree of anisotropy in surface scattering behavior.

ln *ρ* = ln *ρ*_*c*_ + *k* × ln (cos *i*/cos *θ*) (8)

The regression models used for estimating parameters (*C* and *k*) were evaluated using the coefficient of determination (R^2^) to ensure fitting reliability.

#### 2.2.2. Feature Extraction

Single spectral bands are often insufficient to fully characterize the growth status of vegetation canopies, particularly under complex structural and illumination conditions. This study constructed a multi-dimensional feature space by integrating spectral bands, vegetation indices (VIs), and texture features, enabling a comprehensive representation of both physiological and structural characteristics of tea canopies.

Representative canopy spectral information was first extracted from UAV-derived orthomosaics. For each sampled tea plant, all pixels within the canopy projection area were delineated, and the mean reflectance was calculated to characterize canopy-level spectral properties. Based on these spectral data, 16 vegetation indices commonly applied in LAI retrieval were computed (Table 1).

In addition, to quantify canopy structural heterogeneity and leaf spatial arrangement, texture features were extracted using the Gray-Level Co-occurrence Matrix (GLCM) method [50]. To optimize the capture of inter-row and intra-canopy variations, texture metrics were calculated using a 3 × 3 sliding window with a 64-level quantization. The average of four directions (0°, 45°, 90°, and 135°) was used to ensure directional invariance. Specifically, eight texture metrics (Mean, Variance, Homogeneity, Contrast, Dissimilarity, Entropy, Second Moment, and Correlation) were derived from each multispectral band.

In total, 61 feature parameters were generated, including five original spectral bands, 16 vegetation indices, and 40 texture features (eight per band). To eliminate scale-related disparities among features and enhance numerical stability during model training, all features were normalized to a range of [−1, 1].

#### 2.2.3. LAI Inversion Models

To evaluate the applicability and predictive performance of different algorithms for LAI inversion in tea plantations, a comparative analysis was conducted between a traditional linear baseline and three non-linear machine learning models: Linear Regression (LR), Decision Tree (DT), Random Forest (RF), and Extreme Gradient Boosting (XGBoost). These models were constructed based on the extracted multi-dimensional feature set, and their LAI inversion performances were systematically compared to assess their suitability for capturing the complex relationships between canopy characteristics and LAI. All models were implemented using the Python libraries scikit-learn (version 1.8.0) and XGBoost (version 3.2.0).

(1) Linear Regression

As a fundamental statistical baseline, Linear Regression models the relationship between predictor variables and the response variable by estimating regression coefficients using the least squares method. The model assumes a linear relationship between the input features and the target variable (LAI), where the coefficients are obtained by minimizing the sum of squared residuals between observed and predicted values [51]. The prediction function can be expressed as:

(9) $$f(x)=\beta_{0}+\sum_{i=1}^{n}\beta_{i}x_{i}$$

where f(x) denotes the predicted LAI value, $\beta_{0}$ is the intercept term, $\beta_{i}$ represents the regression coefficient of the i-th predictor, $x_{i}$ is the corresponding input feature, and n is the number of predictor variables.

(2) Decision Tree Regression

Decision Tree Regression recursively partitions the feature space into a set of disjoint regions with relatively homogeneous response values. For each leaf node, the predicted LAI is computed as the mean of the training samples within that region. The resulting tree structure explicitly represents a hierarchy of decision rules, facilitating transparent interpretation of how predictor variables contribute to LAI estimation [52]. The prediction function can be expressed as:

(10) $$f(x)=\sum_{m=1}^{M}c_{m}I(x\in R_{m})$$

where *R_m_* denotes the region corresponding to the m-th leaf node, $c_{m}$ represents the mean LAI value of training samples within *R_m_*, M is the total number of leaf nodes, and I(·) represents the indicator function, equal to 1 when x belongs to *R_m_* or otherwise 0.

(3) Random Forest Regression

Random Forest Regression constructs an ensemble of decision trees by repeatedly drawing bootstrap samples from the training data and introducing randomness in feature selection during node splitting. The final LAI prediction is obtained by averaging the outputs of all individual trees. This ensemble structure reduces the sensitivity of the model to variations in the training data and provides a stable mapping between predictor variables and LAI responses [53]. The ensemble prediction is formulated as:

(11) $$f(x)=\frac{1}{M}\sum_{m=1}^{M}T_{m}(x;\Theta_{m})$$

where $T_{m}(x;\Theta_{m})$ represents the prediction of the m-th decision tree, and $\Theta_{m}$ denotes the corresponding randomly sampled parameters.

(4) XGBoost Regression

XGBoost Regression builds an additive ensemble of decision trees in a stage-wise manner, where each successive tree is trained to model the residuals of the current ensemble. Model optimization is performed under a regularized objective function that incorporates gradient-based information. This formulation allows the ensemble to progressively refine the relationship between predictor variables and LAI [54]. The additive prediction model is defined as:

(12) $$f(x)=\sum_{m=1}^{M}{\eta \cdot T}_{m}(x;\Theta_{m})$$

where η is the learning rate that controls the contribution of each individual tree, and the parameters $\Theta_{m}$ of the m-th tree are optimized based on the residuals generated by the preceding m-1 trees.

Hyperparameters were optimized using grid search combined with five-fold cross-validation to ensure robust model tuning and prevent overfitting, with separate optimization performed for terrain-uncorrected and corrected datasets to account for differences in radiometric conditions. The optimal hyperparameters for the machine learning models are summarized in Table 2.

### 2.3. Accuracy Evaluation Metrics

To assess the generalization capability and robustness of the LR, DT, RF, and XGBoost models for tea LAI inversion, a five-fold cross-validation strategy was applied to the entire dataset. Considering the relatively limited sample size typical of field-based LAI measurements, cross-validation was adopted to maximize data utilization while maintaining an independent evaluation framework. The full dataset was randomly partitioned into five mutually exclusive subsets of approximately equal size. In each iteration, four subsets were used for model training, while the remaining subset served as the held-out fold for performance evaluation. This procedure was repeated five times so that each sample was used once for evaluation and four times for training.

Model performance was evaluated using the coefficient of determination (R^2^), root mean square error (RMSE), normalized root mean square error (nRMSE), mean absolute error (MAE), and bias. The R^2^ metric quantifies the proportion of variance in observed LAI explained by the model. RMSE reflects the average magnitude of prediction errors between observed and predicted LAI values, while MAE provides a more robust measure of the average error magnitude that is less sensitive to extreme values. To provide a scale-independent measure of relative error, RMSE was further normalized by the range of observed LAI values and expressed as a percentage (nRMSE). Bias was used to quantify systematic deviation, indicating whether the model tends to overestimate or underestimate LAI. For each model, the reported performance metrics represent the mean values across the five cross-validation folds, providing a robust estimate of predictive accuracy and generalization performance.

### 2.4. SHAP Interpretability Analysis

Shapley Additive exPlanations (SHAP) [27] is a model-agnostic interpretability framework derived from cooperative game theory, which quantifies the marginal contribution of each input feature to an individual model prediction. By decomposing the predicted output into an additive sum of feature attribution values, SHAP enables transparent interpretation of complex nonlinear machine learning models. In this study, SHAP analysis was applied to the best-performing LAI inversion model to investigate both global feature importance and local feature effects. The SHAP framework represents the model prediction as:

(13) $$f(x)=\phi_{0}+\sum_{i=1}^{N}\phi_{i}$$

where $\phi_{0}$ is the expected model output, and $\phi_{i}$ represents the contribution of the i-th feature.

## 3. Results

### 3.1. Topographic Correction Effects

To quantitatively evaluate the effectiveness of the three topographic correction algorithms, their performance was assessed from three complementary perspectives: (i) changes in band-wise mean reflectance, (ii) variations in standard deviation (Std), and (iii) the strength of the linear relationship between reflectance and the cosine of the solar incidence angle(cosi). An effective correction should preserve band-wise mean reflectance, reduce standard deviation by enhancing radiometric homogeneity, and weaken the dependence of reflectance on terrain-induced illumination geometry. Mean reflectance and standard deviation were calculated based on all valid pixels within the sampling plots, ensuring statistical representativeness across different terrain conditions. Beyond observing absolute numerical shifts, paired-sample *t*-tests (*p* < 0.05) and F-tests (*p* < 0.05) were employed to validate the statistical significance of observed changes.

**Figure 5:**
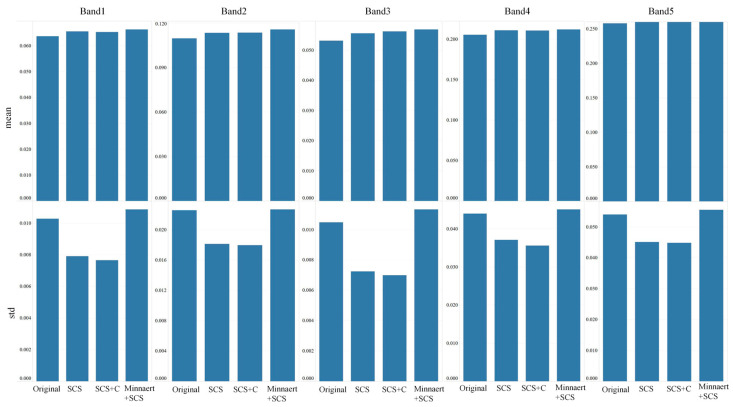
Changes in mean reflectance and standard deviation induced by topographic correction.

As summarized in Table 3 and illustrated in Figure 5, all three correction algorithms produced statistically significant (*p* < 0.05) increases in mean reflectance across all spectral bands. Relative to the original imagery, mean reflectance increased by 2.4–4.7% under SCS, 2.2–5.8% under SCS+C, and 2.7–6.9% under Minnaert+SCS, with the largest relative increases observed in the visible bands (Bands 1–3). Statistical validation confirms that these increments represent a systematic compensation for terrain-induced illumination deficits, effectively mitigating shading effects and enhancing the overall radiometric brightness of the imagery.

After SCS and SCS+C correction, the Std values consistently decreased, with average reductions of 21.2% for SCS and 23.1% for SCS+C, indicating that terrain correction effectively suppressed brightness variability induced by slope and shading, thereby improving spectral consistency within bands. F-tests further confirmed these reductions were statistically significant (*p* < 0.05). SCS+C achieved the largest Std reduction across most bands (Blue: 25.6%, Green: 20.5%, Red: 33.3%, Red-edge: 19.2%, NIR: 17.0%), demonstrating its superior capability in enhancing intra-band homogeneity. In contrast, the Minnaert+SCS correction slightly increased Std values in most bands, with an average increase of 3.8%, suggesting that while shaded areas were brightened, excessive radiometric stretching in sunlit regions led to increased internal spectral heterogeneity.

**Figure 6:**
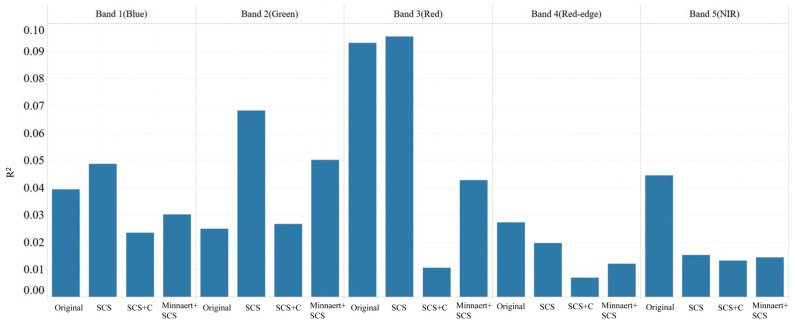
Comparison of R^2^ values from linear regression between cos i and reflectance before and after topographic correction.

The effectiveness of terrain dependency removal was further evaluated by analyzing the coefficient of determination (R^2^) between pixel reflectance and the cosine of the solar incident angle (cos i). As summarized in Table 4 and depicted in Figure 6, all three correction methods reduced R^2^ to varying extents, indicating a weakened correlation between reflectance and illumination geometry. SCS+C achieved the largest reductions in key bands, particularly red band (−88.6%), red-edge band (−74.6%), and near-infrared band (−70.0%), demonstrating its strong capability to mitigate topographic radiometric effects. In comparison, the SCS method exhibited higher R^2^ values than the original imagery in the visible bands, suggesting localized over-correction or the introduction of secondary biases. Although the Minnaert+SCS approach also reduced the correlation between reflectance and topographic factors, its correction exhibited clear radiometric imbalance. Excessive brightening of shaded slopes and darkening of sunlit slopes disrupted spatial radiometric continuity, amplifying reflectance differences among pixels with similar surface properties and thereby compromising correction stability.

Overall, the SCS+C algorithm demonstrated the best comprehensive performance. For low-stature vegetation such as tea plantations, which are characterized by complex terrain and a substantial contribution of diffuse radiation, SCS+C effectively balances direct and diffuse illumination components. This results in improved radiometric consistency across heterogeneous terrain, providing a more stable and physically reliable input for subsequent LAI inversion.

### 3.2. Accuracy Analysis of LAI Inversion Models

**Figure 7:**
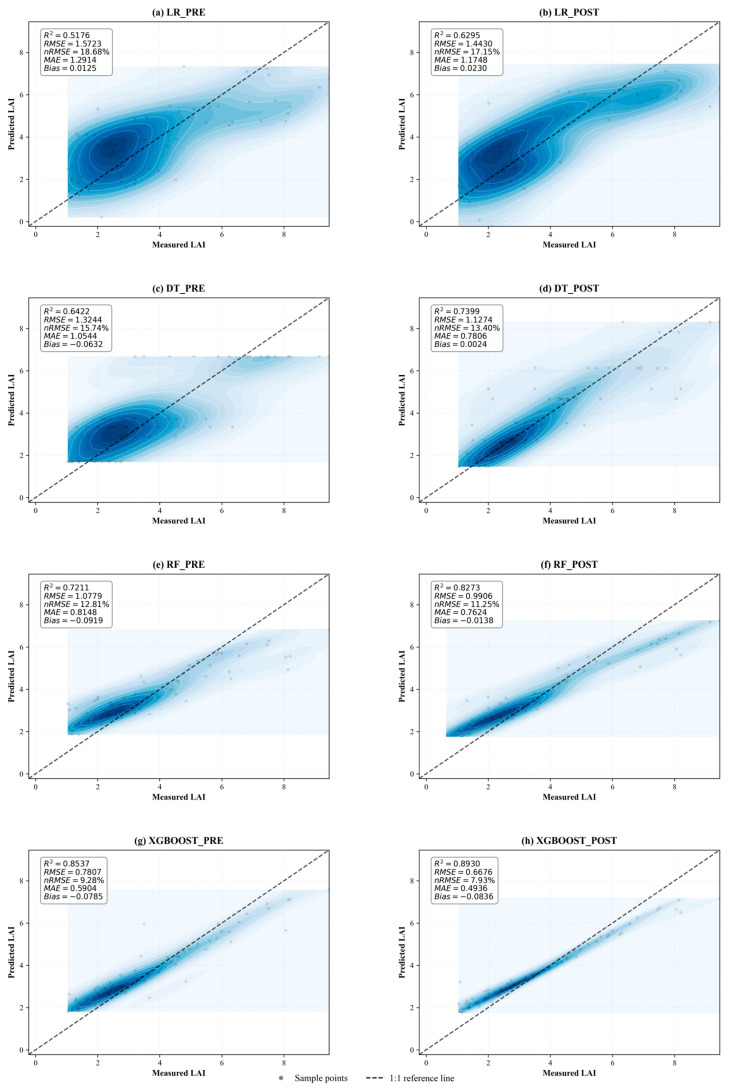
Model performance evaluation results before and after topographic correction.

The performance evaluation results of each inversion model before and after terrain correction are shown in Figure 7. Overall, consistent improvements were observed across all models, in terms of R^2^, RMSE, nRMSE, and MAE, accompanied by bias values generally approaching zero. These improvements indicate that terrain normalization effectively mitigated illumination heterogeneity induced by complex topography, thereby enhancing the consistency between spectral features and canopy biophysical characteristics.

From a model perspective, LR exhibited the lowest predictive accuracy, primarily due to its inherent assumption of linear relationships between spectral variables and LAI, which is insufficient to represent the nonlinear interactions commonly observed in vegetation reflectance, particularly under complex terrain conditions. Before correction, LR achieved an R^2^ of 0.518 and an nRMSE of 18.68%, which improved to 0.630 (+21.62%), and 17.15% (−1.53 percentage points) after correction, respectively. RMSE and MAE also decreased from 1.572 to 1.443 (−8.22%) and from 1.291 to 1.175 (−9.03%). Bias remained slightly positive (from 0.013 to 0.023), indicating a mild tendency toward overestimation. Despite its simplicity, the improvement of LR demonstrates that the effectiveness of topographic correction is not dependent on model complexity but primarily arises from improved data quality.

Compared with LR, nonlinear models showed substantially higher accuracy. Among the machine learning models, the DT model demonstrated the lowest predictive accuracy before topographic correction (R^2^ = 0.642, nRMSE = 15.74%). However, it exhibited the largest relative improvement after topographic correction, with R^2^ increasing to 0.740 (+15.21%), accompanied by reductions in RMSE (from 1.324 to 1.127, −14.87%), nRMSE (−2.34 percentage points), and MAE (from 1.054 to 0.781, −25.97%). Notably, bias shifted from −0.063 to 0.002, indicating that the systematic underestimation present before correction was effectively eliminated. This suggests that DT is particularly sensitive to terrain-induced radiometric noise.

The RF model achieved moderate-to-high accuracy before correction (R^2^ = 0.721, nRMSE = 12.81%) and showed a comparable magnitude of improvement after correction. R^2^ increased to 0.827 (+14.73%), nRMSE declined by 1.56 percentage points, and RMSE and MAE decreased from 1.078 to 0.991 (−8.10%) and from 0.815 to 0.762 (−6.43%), respectively. Although bias remained negative (from −0.092 to −0.014), the magnitude of underestimation was clearly reduced, indicating improved systematic performance. This result suggests that while RF partially mitigates data variability through ensemble learning, it still benefits from improved radiometric consistency after correction.

XGBoost consistently achieved the highest prediction accuracy both before and after correction. R^2^ increased from 0.854 to 0.893 (+4.60%), while RMSE decreased from 0.781 to 0.668 (−14.49%), nRMSE declined from 9.28% to 7.93% (−1.35 percentage points) and MAE decreased from 0.590 to 0.494 (−16.4%). However, bias remained consistently negative (from −0.079 to −0.084), suggesting a slight underestimation tendency that may be associated with spectral saturation effects in high-LAI conditions. While the relative improvement in R^2^ for XGBoost was smaller than that observed for DT and RF, it consistently maintained the lowest overall error levels. This suggests that XGBoost provides more stable predictions across varying radiometric conditions.

Overall, the performance ranking remained consistent across correction conditions (XGBoost > RF > DT > LR). The simultaneous reduction in RMSE and MAE, coupled with mean bias values approaching zero, demonstrates that topographic correction effectively mitigates both random errors and systematic radiometric deviations. Notably, the consistent precision gains observed across models of varying complexity confirm that the benefits of terrain normalization are generalizable and independent of the specific modeling architecture. These findings suggest that topographic correction significantly enhances retrieval consistency, providing a more robust and physically sound basis for LAI inversion in complex mountainous environments.

### 3.3. Interpretability Analysis via SHAP

To elucidate the driving mechanisms and feature contributions underlying the optimal XGBoost-based LAI inversion model, the SHAP method was employed to systematically decompose model prediction behavior and quantify the marginal contribution of individual input variables.

**Figure 8:**
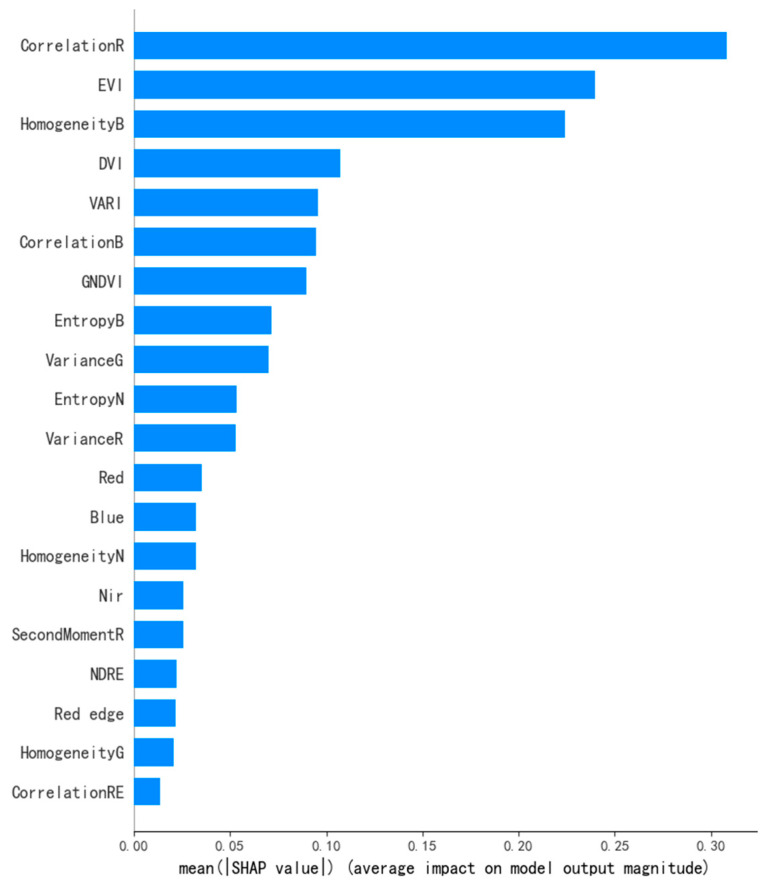
SHAP feature importance plot.

The global SHAP importance ranking (Figure 8) reveals a clear hierarchy in model dependence on input features. Among all variables, the red-band texture feature CorrelationR exhibited the highest mean absolute SHAP contribution (mean |SHAP value| > 0.3), emerging as the most influential predictor of LAI. Other highly ranked contributors were predominantly texture metrics, including HomogeneityB, CorrelationB, EntropyB, VarianceG, and EntropyN. These texture features characterize intra-canopy spatial heterogeneity, shadow distribution, and localized structural variability, providing an explicit representation of canopy spatial architecture at the individual plant scale. Their dominant explanatory power indicates that ultra-high-resolution imagery effectively captures fine-scale canopy structural information critical for LAI estimation. Vegetation indices such as EVI, DVI, VARI, and GNDVI also exhibited substantial contributions, reflecting canopy physiological status and photosynthetic capacity. In contrast, individual spectral reflectance bands (e.g., red, blue, near-infrared, and red-edge) showed comparatively minor contributions. This suggests that the model primarily relies on higher-level feature transformations rather than raw spectral inputs to establish robust nonlinear mappings with LAI.

**Figure 9:**
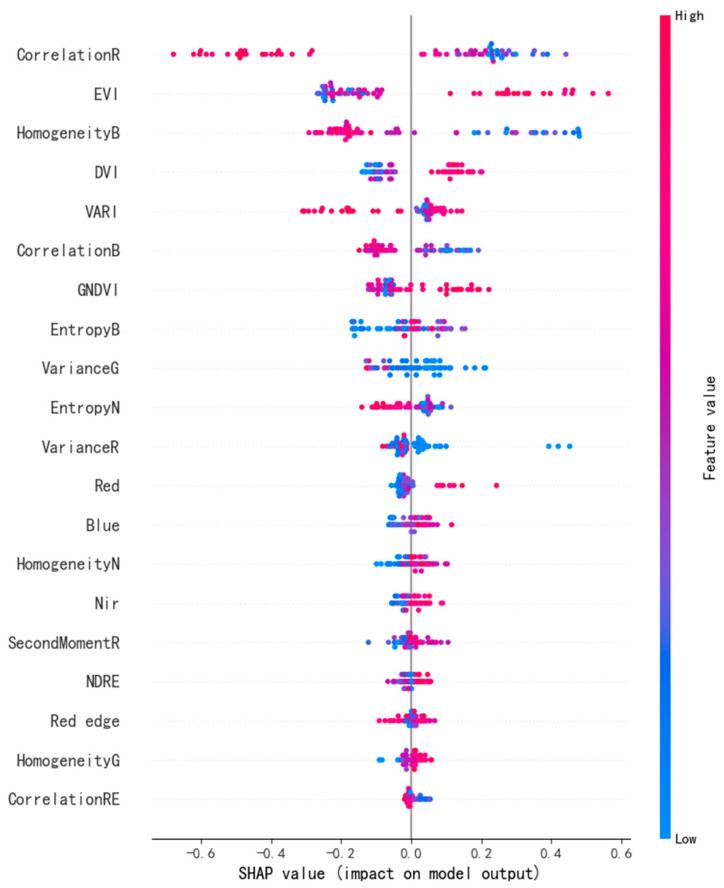
SHAP summary plot.

The SHAP summary plot (Figure 9) further clarifies how variations in dominant features contribute to LAI predictions. The texture feature CorrelationR exhibited the strongest negative relationship with LAI: high feature values (red points) were concentrated in the negative SHAP region, whereas low values (blue points) corresponded to positive SHAP contributions. From a canopy structural perspective, low-LAI conditions, such as early growth stages or sparsely planted areas, are characterized by greater soil background exposure or relatively planar leaf distributions, resulting in higher pixel-to-pixel uniformity and elevated texture correlation. As LAI increases and canopy closure intensifies, enhanced leaf overlap and self-shadowing effects introduce greater brightness discontinuities and shadow patches, reducing gray-level correlation between neighboring pixels. Consequently, lower CorrelationR values serve as reliable indicators of structurally complex, high-LAI canopies. A similar negative relationship was observed for HomogeneityB, where reduced blue-band texture homogeneity reflects increased canopy structural complexity associated with higher LAI. In contrast, vegetation indices such as EVI and DVI exhibited a clear positive driving effect on LAI prediction. High EVI values contributed positively to model outputs, consistent with plant physiological mechanisms related to elevated chlorophyll content and strong multiple scattering in the near-infrared region. These patterns confirm the biophysical plausibility of the model from a spectral perspective.

Overall, SHAP-based interpretation indicates that the optimal XGBoost model adopts a “structure-dominant, spectrum-auxiliary” prediction strategy for LAI inversion. In tea plantations characterized by row-based planting and complex topography, single spectral variables alone are insufficient to characterize complex canopy growth stages. Instead, the synergistic integration of spectral indices and texture features provides a comprehensive and physically interpretable representation of LAI. This complementary mechanism not only validates the rationality of the feature construction strategy adopted in this study but also offers a robust and interpretable framework for precision monitoring of tea plantation growth in topographically complex hilly and mountainous regions.

## 4. Discussion

### 4.1. Applicability of Topographic Correction in Mountainous Tea Plantations

Topographic effects represent a critical source of uncertainty in UAV-based quantitative remote sensing over mountainous agricultural systems. In tea plantations, complex terrain conditions introduce strong spatial variability in illumination, which can ultimately affect LAI inversion accuracy, particularly under high spatial resolution UAV observations [26,55]. The comparative evaluation indicates that the SCS+C algorithm exhibits greater correction stability under such conditions. This improvement can be attributed to its semi-empirical compensation of diffuse irradiance. In subtropical tea-growing regions characterized by dense canopy structure and frequent atmospheric scattering, diffuse radiation may constitute a substantial proportion of total irradiance, especially on partially shaded slopes and between planting rows. By partially balancing the radiometric differences between sunlit and shaded surfaces, SCS+C enhances reflectance consistency across varying terrain conditions.

In contrast, purely geometry-based models (e.g., SCS) may remain sensitive to canopy anisotropy and variations in shadow fraction, particularly in row-structured perennial cropping systems. This is because such models primarily account for direct illumination geometry while neglecting diffuse radiation contributions and directional reflectance effects, leading to incomplete normalization under heterogeneous illumination conditions.

The Minnaert+SCS approach partially alleviates this limitation by introducing an empirical parameter k to account for non-Lambertian scattering behavior. However, this correction is essentially statistical rather than physically based. As a result, while it reduces the dependence between reflectance and illumination geometry, it may introduce increased intra-class variability and does not guarantee physically consistent radiometric normalization across structurally heterogeneous canopies.

Most topographic correction models are developed under the assumption of Lambertian or near-Lambertian surface reflectance. However, tea canopies exhibit pronounced anisotropic reflectance behavior due to their row structure, canopy geometry, and non-uniform shadow distribution. In this study, the Minnaert-based correction was introduced to partially account for anisotropic scattering through the empirical parameter k. Although the Minnaert+SCS model showed improved performance compared to purely geometry-based methods, this improvement should be interpreted as an indirect indication of anisotropy effects rather than a rigorous validation of anisotropic reflectance assumptions. A formal validation of canopy anisotropy, such as through BRDF measurements or multi-angular observations, was beyond the scope of this study.

Overall, these findings indicate that for woody perennial crops characterized by low canopy height, dense canopy structure, and pronounced topographic relief—such as tea plantations—topographic correction models that explicitly account for diffuse radiation components exhibit greater physical robustness and radiometric stability. This study evaluated only three widely used topographic correction algorithms; future research should incorporate BRDF-based correction frameworks or physically based radiative transfer models to explicitly characterize canopy anisotropy and improve the physical interpretability of topographic normalization.

### 4.2. Impact of Topographic Correction on LAI Retrieval Performance

Topographic correction led to consistent improvements in LAI retrieval accuracy across all evaluated models; however, the remaining RMSE, nRMSE, MAE, and bias values indicate that non-negligible uncertainties persist, suggesting that terrain correction alone cannot fully resolve the intrinsic complexity of canopy–radiation interactions.

Residual uncertainties are primarily associated with sub-pixel heterogeneity and canopy structural complexity, which cannot be fully addressed through radiometric normalization. These factors introduce mixed and nonlinear spectral responses that limit the ability of statistical models to uniquely relate reflectance to LAI, particularly under dense canopy conditions where reduced spatial contrast further constrains feature sensitivity.

From a model comparison perspective, all approaches benefited from topographic correction, indicating that its primary role is to improve the consistency and physical interpretability of input features rather than to favor a specific algorithm. The inclusion of Linear Regression as a baseline model further supports this interpretation, as it also exhibited performance improvement after correction despite its limited capacity to capture nonlinear relationships. This suggests that the observed accuracy gains are primarily driven by enhanced data quality rather than increased model complexity.

Differences in model response can be further interpreted from the bias–variance perspective. XGBoost achieved the highest overall accuracy but showed relatively smaller performance gains after correction. As a boosting-based ensemble method, it reduces bias through sequential residual learning while controlling variance via regularization, enabling robust performance even under terrain-induced variability. Consequently, its predictions are relatively stable prior to correction, leaving less room for improvement. In contrast, DT, which is more sensitive to data fluctuations, and RF, which reduces variance through bagging but remains dependent on input quality, benefit more substantially from the reduction of terrain-induced noise.

Overall, these results indicate that topographic correction enhances model performance primarily by reducing radiometric inconsistency and stabilizing feature response relationships, with more pronounced benefits for models that are sensitive to noise and variability. This highlights the importance of data quality as a prerequisite for reliable LAI inversion in complex terrain conditions.

Future improvements in model generalization may require increasing sampling density and expanding coverage across diverse phenological stages and terrain gradients. In addition, further reductions in retrieval uncertainty are likely to depend on integrating physically based radiative transfer constraints, multi-angle observations, or structural descriptors that explicitly capture canopy clumping and vertical heterogeneity, thereby complementing data-driven machine learning approaches.

### 4.3. Structural Dominance Revealed by SHAP-Based Interpretability

The SHAP-based interpretability analysis reveals that the optimal XGBoost model adopts a structure-dominated, spectrum-supported predictive framework. This finding contrasts with previous studies on row crops such as rice and wheat, where vegetation indices (e.g., NDVI and EVI) are typically identified as dominant predictors of crop biophysical parameters [56,57].

This discrepancy is likely attributable to the enhanced spatial heterogeneity induced by the combined effects of row-based tea planting patterns and pronounced topographic relief in mountainous regions. Under such conditions, pixel-level spectral responses are highly susceptible to interference from exposed soil backgrounds and spatially variable illumination. In addition, under relatively high-LAI conditions, traditional vegetation indices tend to exhibit saturation, reducing their sensitivity to incremental changes in canopy density. As a result, spectral variables alone are insufficient to fully capture LAI variability in this context.

In comparison, neighborhood-based texture metrics, which characterize spatial variation rather than absolute reflectance magnitude, are more effective at capturing canopy roughness and spatial structural heterogeneity. This finding is supported by previous studies in forest remote sensing [58,59], which have highlighted the advantage of texture features in representing structural complexity under heterogeneous canopy conditions. In particular, in vegetation systems with pronounced vertical stratification, spectral signals are often subject to saturation and illumination effects, whereas texture features remain sensitive to spatial discontinuities and canopy arrangement, thereby providing enhanced capability for detecting variations in vegetation structure.

Further analysis indicates that texture features derived from the red and blue bands contribute more strongly to LAI prediction than those from the near-infrared (NIR) band. This can be explained by the radiative characteristics of the visible spectrum: strong pigment absorption by leaves, combined with reflectance contrasts between soil and shadow, generates pronounced light–dark variations within the canopy. These contrasts enhance the ability of GLCM metrics to delineate structural boundaries. In particular, the red-band texture feature (Correlation R) appears to effectively capture spatial discontinuities related to canopy gaps, shadow distribution, and row structure, which are closely associated with LAI variation. By contrast, reflectance in the NIR band is dominated by multiple scattering processes, which smooth intra-canopy brightness variations, thereby reducing textural contrast and limiting the structural discriminability of NIR-based texture features.

Overall, the SHAP results indicate that LAI inversion in complex hilly tea plantations is primarily driven by canopy spatial structural heterogeneity, while spectral indices provide essential supplementary information related to vegetation physiology. The synergy between structural and spectral features forms a stable and efficient inversion framework. Future research should examine whether similar feature dominance patterns persist across vegetation types with contrasting canopy architectures and under varying degrees of topographic complexity. Cross-site validation and multi-temporal analysis would further clarify the generalizability of the proposed framework and support the development of physics-informed, transferable LAI inversion models for mountainous agroecosystems.

## 5. Conclusions

This study systematically investigated UAV-based LAI retrieval in tea plantations characterized by complex topography by jointly evaluating topographic correction strategies, machine learning models, and interpretability-based mechanistic analysis. The results demonstrate that terrain-induced illumination heterogeneity constitutes a critical constraint on quantitative agricultural remote sensing in mountainous regions. Among the evaluated correction methods, the SCS+C algorithm achieved superior radiometric consistency across slope aspects by explicitly compensating for diffuse radiation components, thereby exhibiting enhanced physical robustness. Following topographic correction, LAI retrieval accuracy improved consistently across all tested models, with XGBoost demonstrating a distinct advantage in capturing non-linear spectral–structural relationships, with an R^2^ of 0.893, RMSE of 0.668, nRMSE of 7.93%, MAE of 0.494, and Bias of −0.084. SHAP-based interpretability analysis further revealed a structure-dominated retrieval mechanism, in which canopy spatial heterogeneity characterized by textural features plays a primary role, while vegetation indices provide essential supplementary physiological information.

This research advances the process understanding of terrain–radiation–canopy interactions and demonstrates that integrating topographic correction with interpretable ensemble learning offers a robust framework for improving crop monitoring in complex mountainous environments. The proposed methodology provides a solid reference for refined management and precision monitoring of agricultural ecosystems in topographically heterogeneous regions.

However, it should be noted that the proposed framework relies on high-resolution digital elevation models (DEMs) to accurately characterize terrain-induced illumination effects, which may limit its applicability in regions where such data are unavailable or of lower quality. In addition, the model was developed based on a single study area, and its generalizability to other crop types or environmental conditions requires further validation. Future research should focus on improving the adaptability of the approach under varying data availability and extending its application across diverse agroecosystems.

## Figures and Tables

**Table 1:** VIs and formula.

Vegetation Index	Abbreviations	Formula	Reference
Simple Ratio	SR	NIR/R	[34]
Difference Vegetation Index	DVI	NIR − R	[35]
Normalized Difference Vegetation Index	NDVI	(NIR − R)/(NIR + R)	[36]
Normalized Difference Red Edge Index	NDRE	(NIR − RE)/(NIR + RE)	[37]
Soil-Adjusted Vegetation Index	SAVI	(1 + L) × (NIR − R)/(NIR + R + L), L = 0.5	[38]
Enhanced Vegetation Index	EVI	g × (NIR − R)/(NIR + C_1_ × R − C_2_ × B + L), g = 2.5, C_1_ = 6.0, C_2_ = 7.5, L = 1	[39]
Optimized Soil-Adjusted Vegetation Index	OSAVI	(NIR − R)/(NIR + R + 0.16)	[40]
Triangular Vegetation Index	TriVI	0.5 × (120 × (NIR − G) − 200 × (R − G))	[41]
Green Normalized Difference Vegetation Index	GNDVI	(NIR − G)/(NIR + G)	[42]
Green-Blue Normalized Difference Vegetation Index	GBNDVI	(NIR − (G + B))/(NIR + (G + B))	[43]
Visible Atmospherically Resistant Index	VARI	(G − R)/(G + R − B)	[44]
Red–Green–Blue Vegetation Index	RGBVI	(G^2^ − B × R)/(G^2^ + B × R)	[45]
Green Leaf Index	GLI	(2 × G − R − B)/(2 × G + R + B)	[46]
Non-Linear Vegetation Index	NLI	(NIR^2^ − R)/(NIR^2^ + R)	[47]
Modified Triangular Vegetation Index 2	MTVI2	(1.5 × (1.2 × (NIR − G) − 2.5 × (R − G)))/(((2 × NIR + 1)^2^ − (6 × NIR − 5 × R^0.5^) − 0.5)^0.5^)	[48]
Modified Simple Ratio	MSR	(NIR/R − 1)/((NIR/R + 1)^0.5^)	[49]

**Table 2:** Optimized hyperparameter settings for the machine learning models.

Model	Parameter	Uncorrected Imagery	Corrected Imagery
DT	max_depth	10	10
	min_samples_split	5	10
	min_samples_leaf	10	5
RF	n_estimators	200	200
	max_features	Log2	sqrt
	min_samples_split	2	5
	min_samples_leaf	5	5
XGBoost	n_estimators	200	200
	max_depth	5	7
	learning_rate	0.01	0.1
	subsample	0.9	0.8

**Table 3:** Mean reflectance and standard deviation before and after topographic correction.

Band	Original		SCS		SCS+C		Minnaert+SCS	
	Mean	Std	Mean	Std	Mean	Std	Mean	Std
Band 1(Blue)	0.06373	0.01028	0.06555 *	0.00790 *	0.06537 *	0.00765 *	0.06628 *	0.01086
Band 2(Green)	0.10965	0.02259	0.11335 *	0.01813 *	0.11351 *	0.01796 *	0.11556 *	0.02269
Band 3(Red)	0.05311	0.01048	0.05561 *	0.00724 *	0.05619 *	0.00699 *	0.05682 *	0.01133
Band 4(Red-edge)	0.20555	0.04404	0.21114 *	0.03714 *	0.21091 *	0.03559 *	0.21197 *	0.04511
Band 5(NIR)	0.25790	0.05405	0.26415 *	0.04510 *	0.26366 *	0.04487 *	0.26486 *	0.05554

* *p* < 0.05.

**Table 4:** R^2^ values of linear regression between the cosine of solar incident angle and image reflectance.

Band	Original	SCS	SCS+C	Minnaert+SCS
Band 1(Blue)	0.03944	0.04875	0.02345	0.03023
Band 2(Green)	0.02487	0.06824	0.02669	0.05014
Band 3(Red)	0.09300	0.09541	0.01063	0.04279
Band 4(Red-edge)	0.02731	0.01964	0.00695	0.01205
Band 5(NIR)	0.04449	0.01536	0.01332	0.01447

## Data Availability

The data presented in this study are available on request from the corresponding author.
